# Determinants of patient satisfaction with ophthalmic services

**DOI:** 10.1186/1756-0500-4-7

**Published:** 2011-01-15

**Authors:** Hossain Ziaei, Marzieh Katibeh, Armen Eskandari, Monir Mirzadeh, Zahra Rabbanikhah, Mohammad Ali Javadi

**Affiliations:** 1 Ophthalmic Research Center, Labbafinejad Medical Center, Shahid Beheshti University of Medical Sciences, Tehran, Iran

## Abstract

**Background:**

This cross-sectional study was conducted during summer 2008 at an academic ophthalmic hospital to assess patient satisfaction with care services and examine the impact of different dimensions on overall satisfaction.

**Findings:**

Clients of ophthalmic services were selected by systematic random sampling. Overall satisfaction was measured as the primary outcome using a validated patient satisfaction questionnaire (PSQ-18). Different domains were evaluated using PSQ-18 (technical quality, interpersonal manner, communication, financial aspects, time spent for patients, convenience and accessibility); an additional domain, physical setting of the hospital, was evaluated by complementary questions. A general linear model was used to assess the adjusted impact of each quality dimension on the overall satisfaction. Accessibility and technical quality had the strongest association with the overall satisfaction. This regression model could predict an overall satisfaction of 60%.

**Conclusions:**

In comparable settings, if care providers wish to improve the quality of health services from a patients' perspective, they should give priority to improving accessibility and technical quality. Further studies are recommended to discover complementary predictors in formation of overall satisfaction.

**Previous publication:**

Some parts of this article are translated form Farsi originally published in Bina Journal of Ophthalmology. (2009; 14 (3): 289-297). The original work is at: http://binajournal.org/index.php/bina/article/view/96.

## Findings

### Background

Research on patient satisfaction with medical care can be tracked to the late 1960s [1]. Initially, researchers focused on patient satisfaction as an intermediate condition in order to reach desirable clinical outcomes such as patient compliance with recommended treatment [2]. Gradually, patient satisfaction was shifted to a final outcome for evaluating and improving health and care services [3].

Different instruments have been used to measure satisfaction [4-12]. Studies dealing with patient satisfaction are not homogenous and more studies are needed to ascertain the best technique for measuring quality of health care services and the importance of various predictors on overall satisfaction. In addition, little information is available on patient satisfaction in Iran. The overall satisfaction in four social security hospitals in Tehran, in 3017 inpatients in hospitals in Kerman and patient satisfaction among women attending the Iranian Centre for Breast Cancer were around 60%, 50% and 82% respectively [13-15]. The two former studies were performed in general hospitals and the latter in a subspecialty hospital which might justify the difference observed. The present study is unique in that it attempted to assess patient satisfaction with eye care services in Iran and to examine the impact of different dimensions on overall satisfaction.

### Method

The research was conducted during summer 2008 at a main academic hospital (Labbafinejad Medical Center), in Tehran. This hospital has a high turnover of inpatient/outpatient clients, with nearly 16,000 ophthalmologic surgeries, 90,000 outpatient visits, 23,000 patients visiting the emergency department (ED) and 4,000 patients being admitted to hospital wards in the corresponding year.

The study was approved by the ethics Committee of Shahid Beheshti University of Medical Sciences, and oral informed consent was obtained from all participants. Patients of various ethnicities and ages who attended different inpatient/outpatient ophthalmic services were eligible to be assessed, except those who could not mentally or physically communicate. Sixty patients were selected from each section, except from the operation room where 120 patients were selected to cover all operation types properly. Patients were selected from the admission list through systemic random sampling. In order to avoid any selection bias, all patients had an equal chance to enter the study at anytime during their attendance, even those who did not continue their treatment, Using n = [Z2×P× (1-P)/d2)] formula, where d = 0.05 and type I error of 0.05 and considering at least 50% of overall satisfaction based on previous studies in Iran [14,15] and nearly 10% non-response rate, the sample size was calculated at least 410 persons.

The data collection tool was a three- part questionnaire; the first part consisted of demographic questions, patient's evaluation about his/her eye health and insurance status. The second part was based on the standard PSQ-18 questionnaire [5] and the third part consisted of complementary questions regarding physical setting of the hospital. PSQ-18 evaluates such dimensions as technical quality, interpersonal manner, communication (doctor-patient), financial aspects, time spent for patient, convenience, accessibility and overall satisfaction. The questionnaire was completed by a trained interviewer at the time of discharge. The validity of the original PSQ-18 was already verified [5,12]. In this study it was translated to Persian and its content validity was evaluated using Delphi method and a multidisciplinary approach that involved ophthalmologists, social workers, medical staff members of the hospital and patients. The reliability was also checked by a pilot study in a group of 20 patients using a test-re-test assessment; the statistical measure of agreement (Kappa) between individuals' paired responses was more than 0.92.

In addition, 40 patients were asked to answer the questions in order to find the difficulties in performing the investigation and the clarity of the questions. The results of the pilot phase results were not used for final analysis.

### Statistical analysis

Variables were described using mean ± standard deviation (SD) or percentage where appropriate. All the questions that assess satisfaction level were scored on a five-point Likert scale. Cronbach's alpha was used to assess internal consistency of questions in the physical setting dimension. After scoring, items within the same subscale were averaged together to create subscale scores. Univariate and multivariate statistical tests were used to assess and compare the effect of different variables on overall patient satisfaction. In all analyses α < 0.05 was considered as statistically significant. In a regression model, the contribution of individual domains including technical quality, interpersonal manner, communication, financial aspects, time spent for patient, convenience, accessibility (assessed by PSQ-18) and physical setting of the hospital (assessed by complementary questions in our study) to overall satisfaction, as the outcome variable, was addressed. All statistical analyses were done using SPSS-17.

## Result

Among 550 selected patients, 539 completed the questionnaire (response rate = 98%). The mean ± SD age of the responders was 44.7 ± 23 years. Other baseline characteristics are presented in Table 1.

**Table 1:** Baseline characteristics of participants (n = 539)

	number	%
**Sex**		
Male	280	52
Female	259	48
**Residence**		
Urban	391	72
Rural	148	28
**Time of visit**		
First	211	39.3
Second and more	326	60.7
**Education **(n = 459, patients above 18)		
No education	130	28.3
Elementary	125	27.2
Middle school	46	10
High school	98	21.4
Academic	60	13.1

Of selected patients, 167 patients (31.2%) were completely satisfied, 215 (39.9%) were satisfied, 95 (17.5%) were partially satisfied, 29 (5.2%) were unsatisfied, and 33 (6.2%) were completely unsatisfied. The average overall satisfaction score, measured by the PSQ-18 questionnaire, was 4.05 ± 1.1 from a maximum of 5. This score was not statistically different among subgroups of age (p = 0.49), sex (p = 0.36), education (p = 0.93) and location of residence (p = 0.25).

Various dimensions of service quality in different departments of hospital are shown in table 2. As shown in the table, the overall satisfaction was heterogeneous in different departments (p < 0.001) with ED obtaining the lowest total satisfaction score. Moreover; among different quality dimensions, accessibility and convenience received lower scores in all departments.

**Table 2:** Mean values of participants' satisfaction according to PSQ-18 questionnaire

	Different Departments					
	Total (n = 539)	Emergency (n = 59)	Operating room (n = 108)	Ward (n = 62)	Clinics (n = 310)	P value*
Technical quality	4.1 (0.9)	3.1 (0.9)	4.2 (0.8)	4.2 (0.7)	4.1(1)	< 0.001
Interpersonal manner	4.6 (0.7)	4.3 (0.7)	4.7 (0.5)	4.5 (0.8)	4.6 (0.8)	0.015
Communication	4.3 (0.9)	3.8 (1.0)	4.6 (0.6)	4.3 (1.0)	4.4 (0.9)	< 0.001
Financial aspects	4.5 (1.1)	4.6 (1.07)	4.5 (1.0)	4.3 (1.2)	4.5 (1.2)	0.67
Accessibility	3.8(1.1)	3.6 (1.0)	4.3 (0.7)	4.0 (0.8)	3.6 (1.2)	< 0.001
Convenience	2.6 (1.2)	3.2 (0.8)	3.0 (1.3)	2.4 (1)	2.4 (0.9)	< 0.001
Time spent for patient	4.3(0.9)	4.1 (0.9)	4.6(0.5)	4.4(0.6)	4.1(1.0)	< 0.001
Overall satisfaction	4.0 (1.1)	3.5 (1.0)	4.3 (0.7)	4.0 (1.0)	4.0 (1.2)	< 0.001

Data presented are means ± standard deviation and satisfaction level were scored on a five-point Likert scale

**P- Value for difference in satisfaction dimensions by different hospital departments based on ANOVA test

Apart from two patients (0.5%), who had no insurance and 48 patients (9%) with insurances incompatible with hospital paying system (who should pay completely for their care), 411 persons (76.6%) had social security insurance and received totally free services and 31 persons (11.5%) had medical service insurance which covers only half of care costs in this hospital. Although there were no statistically significant difference in overall satisfaction among various insurance types (p = 0.98), a significant difference was noted in the financial aspect of satisfaction among insurance subtypes with a mean score of 4.8 ± 0.7 for social security insurance users, 3.8 ± 1.5 for medical services insurance clients and 3 ± 1.8 for other patients (p < 0.001).

The physical environment of the hospital (including visual environments. quietness, temperature, and cleanliness) received an average satisfaction score of 4.2 ± 0.9. Cronbach's alpha was 0.84 for the four questions that assessed the physical environment.

Regarding self-rated eye health, 19 persons (3.6%) estimated their visual health and performance as poor, 227 persons (42.8%) as intermediate and 284 persons (53.6%) as good. Of those who evaluated their visual health as poor, overall satisfaction was 3.8 ± 1.4. There was no significant difference between this group and those who evaluated their visual health as intermediate (4.03 ± 1.1) or good (4.08 ± 1.02) (p = 0.58). Among various service quality dimensions, technical quality and time spent for patient were statistically different among three grades of self-rated visual health. The average satisfaction levels of technical quality were 4.1 ± 0.9, 4 ± 1 and 3.5 ± 1.2 in patients with good, intermediate and poor vision self-rating, respectively (p = 0.042) and the average satisfaction level of time spent for patient in the three stated grades were 4.3 ± 0.9, 4.3 ± 0.9 and 3.5 ± 1.6 correspondingly (p = 0.043).

Multiple regression was used to examine the correlation between overall satisfaction and other dimensions after adjustment for different hospital departments. The service dimensions that had the strongest association with overall satisfaction in this model were (in order of importance): accessibility (β = 9.2, p < 0.001), technical quality (β = 6.3, p < 0.001), convenience (β = 3.9, p < 0.001), time spent for patient (β = 3, p = 0.001), communication (β = 2.7, p = 0.001), and physical environment (β = 2.4, p = 0.011). This regression model could predict an overall satisfaction of 60% (R square = 0.61).

## Discussion

The formation of overall satisfaction in an individual is related to psychological, cultural and environmental factors which have not been properly identified yet. We do believe that the interaction of factors constituting overall satisfaction is different among cultures; therefore overall satisfaction could not be precisely estimated by simply measuring the statistical average of certain aspects. In the current study, patient satisfaction varied in different dimensions. The highest satisfaction level was related to interpersonal manner and financial aspects and the lowest was related to convenience and accessibility. However; using multiple regression analysis, it was shown that different dimensions of service quality (evaluated by PSQ-18) and physical setting could only predict 60% of overall satisfaction. Besides; it was found that physician accessibility and the technical quality had the strongest correlation with the level of overall satisfaction while physical setting and financial aspects had the weakest. Therefore, in comparable settings, if the managers of health care centers wish to improve the quality of their services from a patients' viewpoint, they should give priority to these aspects.

Client satisfaction might be influenced by social situation and is related with patient expectancy of services. In the current study, patients reported a relatively acceptable level of overall satisfaction which was comparable with the study by Sadjadian and colleagues [13] but higher than two other previous studies in this field in Iran [14,15]. However, none of these studies were conducted in ophthalmic subspecialty hospitals and different tools were used for estimating satisfaction.

In the current study, the mean scores for the convenience and accessibility domains were less than the other aspects; this is in contrast with Jagadeesan's study [9] that used similar tools for quality measurement and scoring method in an eye clinic where ophthalmology residents provided care for patients. Therefore, an opportunity exists to improve this aspect in our setting.

In the present study, there was no statistically significant difference in patient satisfaction among various demographic subgroups; a meta-analysis by Hall and Dornan [16] confirm this finding; however, there is controversy over importance of patients' demographic and social factors in determining satisfaction [17]. This inconsistency in terms of the effect of education on patient's satisfaction was also seen in previously published Iranian papers; while Sadjadian and colleagues [13] showed no significant relationship in capital city, another study on 3017 patients in a rather deprived city demonstrated a statistical deference [15].

Some other predictors of patient satisfaction have been introduced in previous studies; they had the same conclusion as ours in terms of self-rated eye health and insurance type [[10,11] and [15]].

PSQ-18 was designed using 18 questions to ensure rapid completion (2-3 minutes) [5] and has been used in different recent studies [[9,12] and [18]]. In the current study, this questionnaire was translated to Persian and the content validity was assessed. In the original questionnaire, accessibility and convenience were grouped together; however, authors believe these aspects are not well matched in the concept; therefore, they were assigned to separate groups. Since there were no questions in PSQ-18 related to physical environment, authors introduced complementary questions and checked their internal consistency. However, according to our results further predictors influence the formation of overall satisfaction.

## Conclusion

In our experience, accessibility and scientific-technical quality have the strongest association with overall patient satisfaction. Therefore, accessibility and scientific-technical ability should be among managers' high priorities if they wish to improve quality of services from a patients' viewpoint. Different dimensions of service quality evaluated by PSQ-18 and satisfaction of physical setting could only predict 60% of overall satisfaction; as a result, further studies are recommended to discover how overall satisfaction is formed and can be increased in different cultures.

## Competing interests

The authors declare that they have no competing interests.

## Authors' contributions

HZ and MAJ suggested the initial concept, provided administrative and technical support for conducting the study and supervised the data collection. MK and AE contributed to the study design, performed the statistical analysis and interpretation of data and did critical revision of the manuscript for important intellectual content. ZR and MM participated in the data collection, prepared the final report and drafting manuscript. All authors read and approved the final manuscript.
